# Item Reduction, Psychometric and Biometric Properties of the Italian Version of the Body Perception Questionnaire—Short Form (BPQ-SF): The BPQ-22

**DOI:** 10.3390/ijerph18073835

**Published:** 2021-04-06

**Authors:** Andrea Poli, Angelo Giovanni Icro Maremmani, Carlo Chiorri, Gian-Paolo Mazzoni, Graziella Orrù, Jacek Kolacz, Stephen W. Porges, Ciro Conversano, Angelo Gemignani, Mario Miccoli

**Affiliations:** 1 Department of Surgical, Medical and Molecular Pathology and of Critical Care Medicine, University of Pisa, 56126 Pisa, Italy; graziella.orru@unipi.it (G.O.); ciro.conversano@unipi.it (C.C.); angelo.gemignani@unipi.it (A.G.); 2 Department of Clinical and Experimental Medicine, University of Pisa, 56126 Pisa, Italy; mario.miccoli@med.unipi.it; 3 Verdi Clinical Center, 59100 Prato, Italy; 4 Florence Cognitive School, 50144 Florence, Italy; mazzoni.psico@gmail.com; 5 Department of Psychiatry, North-Western Tuscany Region NHS Local Health Unit, Versilia Zone, 55049 Viareggio, Italy; angelo.maremmani@uslnordovest.toscana.it; 6 Association for the Application of Neuroscientific Knowledge to Social Aims (AU-CNS), 55045 Pietrasanta, Italy; 7 G. De Lisio Institute of Behavioral Sciences, 56100 Pisa, Italy; 8 Department of Educational Sciences, University of Genova, 16121 Genova, Italy; carlo.chiorri@unige.it; 9 Traumatic Stress Research Consortium at the Kinsey Institute, Indiana University, Bloomington, IN 47405, USA; jkolacz@iu.edu (J.K.); sporges@indiana.edu (S.W.P.); 10 Department of Psychiatry, University of North Carolina, Chapel Hill, NC 27514, USA

**Keywords:** body perception, body, interoception, polyvagal theory, psychological trauma, vagus nerve

## Abstract

Body awareness disorders and reactivity are mentioned across a range of clinical problems. Constitutional differences in the control of the bodily state are thought to generate a vulnerability to psychological symptoms. Autonomic nervous system dysfunctions have been associated with anxiety, depression, and post-traumatic stress. Though interoception may be a transdiagnostic mechanism promoting the improvement of clinical symptomatology, few psychometrically sound, symptom-independent, self-report measures, informed by brain–body circuits, are available for research and clinical use. We validated the Italian version of the body perception questionnaire (BPQ)—short form and found that response categories could be collapsed from five to three and that the questionnaire retained a three-factor structure with items reduced from 46 to 22 (BPQ-22). The first factor was loaded by body awareness items; the second factor comprised some items from the body awareness scale and some from the subdiaphragmatic reactivity scale (but all related to bloating and digestive issues), and the third factor by supradiaphragmatic reactivity items. The BPQ-22 had sound psychometric properties, good convergent and discriminant validity and test–retest reliability and could be used in clinical and research settings in which the body perception assessment is of interest. Psychometric findings in light of the polyvagal theory are discussed.

## 1. Introduction

Body awareness disorders and reactivity are mentioned across a range of clinical problems, and recently, it has been proposed that vulnerability to psychological symptoms, particularly anxiety, may originate in constitutional differences in the control of the bodily state [1]. Joint hypermobility syndrome, or Ehlers–Danlos syndrome hypermobile type (JHS/EDS-HT), has shown a strong association with anxiety disorders but also with depression, eating, and neuro-developmental disorders as well as alcohol and tobacco misuse [2,3] and JHS/EDS-HT patients scored higher for interoceptive sensitivity [4]. In addition, postural orthostatic tachycardia syndrome patients often exhibit mild to moderate depression and anxiety disorders symptoms [5] as well as vasovagal syncope patients [6]. Though the underlying mechanisms behind this association include genetic risks, autonomic nervous system (ANS) dysfunction, increased exteroceptive and interoceptive mechanisms and decreased proprioception have been associated with anxiety [7,8], depression [9,10], post-traumatic stress [11], autism [12,13], schizophrenia [14], eating disorders [15,16] and in adults with hypertension [17]. Though interoception, or the process of sensing, interpreting and integrating internal bodily signals, has been linked to various clinical conditions and several randomized controlled trials found that interventions with interoception were effective in ameliorating symptoms related to anxiety disorders, eating disorders, psychosomatic disorders, and addictive disorders that also encompassed improvements of interoceptive measurements, measures self-reporting ameliorating symptoms are often related to specific illness symptoms only. Interoception may be a transdiagnostic mechanism of action promoting the improvement of clinical symptomatology; however, few studies include general, symptom-independent interoceptive self-report tools and few psychometrically sound self-report measures, informed by brain–body circuits, are available for research and clinical use. To deepen our knowledge of the role that interoception has in psychological conditions and their treatment, additional research involving the use of interoceptive measures is needed, as well as a clearer statement of interoceptive terminology [18].

Interoceptive awareness emerges from a complex network of afferent and efferent pathways that project from bodily organs and structures to the central nervous system that, in turn, regulate somatic and visceral motility [19,20,21]. These ANS signals are relayed through autonomic pathways that are typically identified in antagonistic dynamics of sympathetic and parasympathetic nervous activities [22,23]. However, current views highlight a multilevel ANS activity that reflects multiple coordinated systems and that can lead to unbalanced autonomic activities, which may contribute to the generation of various disorders, including cardiovascular, inflammatory, metabolic, neurological, and psychiatric diseases [24].

The polyvagal theory [25,26] provides a neurophysiological and evolutionary framework related to autonomic systems’ organization. Relying on evidence stemmed from comparative anatomy, neurophysiology and behavioral observations, this theory identifies two distinct vagal circuits within the parasympathetic nervous system that form a ventral vagal complex (VVC) and a dorsal vagal complex (DVC).

According to the polyvagal theory [25,26], the human autonomic nervous system’s structural organization and function is hierarchically rooted in its phylogenetic heritage. The social engagement system stems from the myelinated ventral vagal complex (VVC), whose cardioinhibitory fibers originate in the nucleus ambiguus (NA) in the brainstem. VVC is a challenge–response system that is the least homeostatically disruptive, the phylogenetically youngest and the most rapidly acting (due to its myelinated fibers). The sympathetic nervous system (SNS) is phylogenetically older than the VVC; its activation promotes faster heart rate, respiration, and mobilization for active threat responses, such as escape or confrontational defense. The dorsal vagal complex (DVC), whose unmyelinated cardioinhibitory fibers originate in the vagus’s dorsal motor nucleus (DMNX) in the brainstem, is the phylogenetically oldest of the autonomic subsystems and includes a vestigial immobilization function that first arose in early vertebrates. DVC is involved in both homeostatic and threat reactions and primarily innervates organs below the diaphragm. This complex also disrupts digestive processes and conserves metabolic resources when recruited during threat responses [25,26]. Interestingly, DVC is the system that is primarily involved in post-traumatic responses following psychological trauma [27,28]. The polyvagal theory proposes that the integration of the myelinated cardiac vagal pathways with the neural regulation of the face and head promoted the emergence of the mammalian social engagement system. The outputs of the social engagement system consist of motor pathways regulating striated muscles of the face and head (i.e., somatomotor) and smooth and cardiac muscles of the heart and bronchi (i.e., visceromotor). The somatomotor component involves special visceral efferent pathways that regulate the striated muscles of the face and head. The visceromotor component involves the myelinated supradiaphragmatic vagal pathway that regulates the heart and bronchi. Functionally, the social engagement system emerges from a face–heart connection that coordinates the heart with the muscles of the face and head. The initial function of the system is to coordinate sucking, swallowing, breathing, and vocalizing. Atypical coordination of this system early in life is an indicator of subsequent difficulties in social behavior and emotional regulation. The preferential recruitability of the social engagement system, or the progressive hierarchical recruitment of the SNS or the DVC, depends on the neural evaluation of environmental risk. According to the polyvagal theory, the neural evaluation of risk does not require conscious awareness and is achieved through neuroception, a neural reflexive mechanism capable of instantaneously shifting physiological state, distinct from perception, and capable of distinguishing environmental and visceral features that are safe, dangerous, or life-threatening. In safe environments, a neuroception of safety promotes the social engagement system and the autonomic state is adaptively regulated to dampen SNS activation and to protect the oxygen-dependent central nervous system, especially the cortex, from the metabolically conservative reactions of the DVC (e.g., fainting). Conversely, a neuroception of danger, or life threat, promotes SNS, or DVC, activation, respectively [25,26,29]. The organization of these individual circuits, along with the sympathetic nervous system, can affect subjective experiences of body awareness by modulation of signals that arise from the body by top-down postprocessing, including cortical areas informed by the information traveling through the body-integrative circuits of the brain [19].

Several tools have been developed to assess interoception but, following the review of Mehling and colleagues [21], they do not address important domains of the construct of body awareness and exhibit important psychometric limitations. To address these limitations, new psychometrically sound tools have been developed (e.g., [30,31,32]). Unfortunately, the self-report measures that show sound psychometric properties have not been rooted in the organization of peripheral neural pathways.

The body perception questionnaire (BPQ; [33]) was developed to evaluate the subjective experience of the function and reactivity of bodily organs and structures that are innervated by the ANS. The first version of BPQ, consisting of 122 items, assessed body awareness, ANS reactivity, cognitive–emotional–somatic stress response, body and cognitive stress response styles, and health history. Though the original BPQ has been widely used in several peer-reviewed publications and translated into several languages, its lack of psychometric testing and its extensive length has limited its broader application. The interest in BPQ has been mainly related to body awareness and autonomic reactivity subscales. Past research has mainly used only these subscales (e.g., [34,35]). In order to overcome these limitations, Cabrera and colleagues [36] validated the BPQ-short form (BPQ-SF) and found that body awareness was described by a single factor. In addition, autonomic reactivity reflected unique factors for organs above and below the diaphragm. Subscales showed strong reliability and converged with validation measures.

A paucity of self-report measures to assess body awareness and somatic reactivity is validated in the Italian language. The somatosensory amplification scale (SSAS; [37]; Italian version in Bernini et al. [38]) was originally developed to assess somatosensory amplification (SA), defined as the individual’s tendency to experience somatic and visceral sensations as unusually intense, noxious, and disturbing. SA has been proposed to be a risk and/or maintenance factor for hypochondriasis, somatization, and, in general, physical symptom reports. The modified somatic perception questionnaire (MSPQ; [39,40] was originally developed for investigating chronic backache, or other forms of chronic pain, stroke and cardiovascular diseases, tinnitus and Meniere’s disease and patients undergoing surgery. It has also been used to measure somatization in nonpainful conditions. Furthermore, the Italian version of the MSPQ [41] has been used with immigrant populations [42]. Though informative, unfortunately SSAS and MSPQ are not rooted in a neurophysiologically informed and reliable background.

Considering the lack of a psychometrically sound tool aimed to assess body awareness and supradiaphragmatic and subdiaphragmatic autonomic reactivity, according to the polyvagal theory, validated in the Italian population, this paper aims to examine the psychometric properties and validate the BPQ-SF [36] to provide a useful tool that can be employed in both clinical and research fields. In particular, the present study aims to: (a) examine the factor structure and psychometric properties of the BPQ-SF among a non-clinical sample; (b) examine the BPQ-SF internal consistency and test–retest reliability; (c) demonstrate convergent validity with the SSAS, the MDSP and the depression anxiety stress scales-21 (DASS-21; [43,44,45]; Italian version in Bottesi et al. [46]; (d) demonstrate a discriminant validity with the obsessive beliefs questionnaire—20 (OBQ-20; [47]; Italian version in Melli et al. [48]).

## 2. Materials and Methods

### 2.1. Participants

We collected two samples of participants. The first sample consisted of 1.361 (80.9% female) community participants (M = 37.29 years, SD = 9.94 range 18–81), who responded to an email advertisement requesting volunteers to complete psychological questionnaires. In terms of education, 3.97% of the participants had a medium-level of education (high school degree), 40.41% had a higher-level degree (bachelor’s degree or master’s degree), and the remaining 55.62% had the highest level of education (Ph.D. or specialization). Most were employed (89.93%), 3.53% were undergraduate university students, and the remaining 6.54% were homemakers, unemployed, or retired. Regarding marital status, 47.98% were single, 46.51% were married or cohabiting, 4.78% were divorced, and 0.73% were widows or widowers. These participants completed a battery of measures described in the next section.

A second sample of 97 (84.7% female, mean age 37.08 years, SD = 10.17, range 24–67) participants, who completed the BPQ-SF twice at a 3 week interval, was recruited with the same strategy as the first. They had a master’s degree or higher, and the majority was employed (90.82%). Sixty percent of them were single or divorced, with the others having a stable relationship.

To be included in the study, participants must be 18 or older and not report any history of psychiatric or psychological disorders.

### 2.2. Measures

*Body Perception Questionnaire—Short Form (BPQ-SF; [36])*. The BPQ is a self-report measure of body awareness and autonomic reactivity originally developed by Porges [33] and then refined by Cabrera et al. [36]. Although the latter authors introduced a very brief, 12-item version, we focused here on the 46-item version reported in the BPQ manual [49]. Items ask participants to rate on a 5-point scale (from 1 = *never* to 5 = *always*) the frequency with which they feel aware of bodily sensations (body awareness, e.g., “My mouth being dry”) and the frequency with which they experience supradiaphragmatic reactivity (e.g., “I feel shortness of breath”), and subdiaphragmatic reactivity (e.g., “I have indigestion”).

The Italian translation of the BPQ-SF was obtained through a mixed forward- and back-translation procedure [50]. The authors and one bilingual Italian–English psychologist independently translated the English version of the scale into Italian. After consensus among translators was achieved, an Italian–English researcher blind to the original version translated this preliminary version back into English. Discrepancies emerging from this back-translation were discussed with the original authors of the scale. Before being used in this study, the newly developed Italian version of the BPQ-SF was administered to ten participants (not included in the present study) in order to check the understandability of the items, which were all found to be easy to understand and to provide ratings for.

*Somatosensory Amplification Scale (SSAS; [37])*. The SSAS is a measure of “somatosensory amplification”, i.e., the individual’s tendency to experience somatic and visceral sensations as unusually intense, noxious, and disturbing. It comprises 10 items that ask the participant to report how much she is bothered by various uncomfortable visceral and somatic sensations that, however, are not pathological symptoms of serious diseases. In this study, we used the Italian version for the SSAS developed by [38].

*Modified Somatic Perception Questionnaire (MSPQ; [39,40])*. The MSPQ is a list of 22 symptoms of heightened somatic awareness (e.g., “feeling hot all over”, “blurring of vision”). Items are rated on a 4-point scale of severity (1 = not at all, 4 = “extremely, could not have been worse”), and the total score is derived from the sum of the original 13 items introduced by [39]. In this study, we used the Italian version by [41].

*Depression Anxiety Stress Scales–21 (DASS-21; [43,44,45])*. The DASS-21 is a 21-item self-report questionnaire that assesses the core symptoms of depression (including lack of incentive, low self-esteem, and dysphoria, e.g., “I couldn’t seem to experience any positive feeling at all”), anxiety (including somatic and subjective symptoms of anxiety, as well as acute responses of fear, e.g., “I felt scared without any good reason”), and stress (including irritability, nervous tension, difficulty relaxing, and agitation, e.g., “I tended to over-react to situations”). Participants are asked to rate the severity of the symptoms over the past week on a 4-point scale (1 = “Did not apply to me at all”, 4 = “Applied to me very much or most of the time”). In this study, we used the Italian version by [46].

*Obsessive Beliefs Questionnaire-20 (OBQ-20; [47])*. The OBQ-20 is a 20-item version of the original 87-item [51] 2001 and the subsequent 44-item version [52]. The purpose of this scale is the assessment of four dysfunctional belief domains that can lead to a misappraisal of intrusive thoughts: (i) threat (e.g., “If I do not take extra precautions, I am more likely than others to have or cause a serious disaster”); (ii) inflated responsibility (e.g., “If I don’t act when I foresee danger, then I am to blame for consequences”); (iii) importance and control of thoughts (also assesses need to control thoughts; e.g., “For me, having bad urges is as bad as actually carrying them out”); and (iv) perfectionism (also assesses intolerance of uncertainty; e.g., “I must keep working until it’s done exactly right”). Items are rated on a 7-point agreement scale (1 = “disagree very much”, 7 = “agree very much”). The Italian version we used here is the one by [48].

### 2.3. Procedure

The questionnaires were made available online using a secure web-based survey program (SurveyMonkey). Questionnaires were administered in a counterbalanced fashion to control for order and sequence effects, and batteries took between 15 and 25 min to complete. All participants volunteered to take part in the study after being presented with a detailed description of the procedure and were treated in accordance with the Ethical Principles of Psychologists and Code of Conduct [53]. No external incentives were offered for participating in this study.

### 2.4. Data Analysis

As a first step in the analyses, we examined the item score distributions in order to evaluate the frequency distributions of the scores for each item, namely, whether all the values of the answer scale had been endorsed at least once, and we also assessed the extent of the missing data. Shapiro–Wilk test was performed to verify the normality of the distributions.

We then tested on the total sample through confirmatory factor analysis (CFA) using the weighted least squares with means and variance adjustment (WLSMV) estimator (theta parameterization) whether the hypothesized 3-factor structure (body awareness, supradiaphragmatic reactivity, and subdiaphragmatic reactivity, taking into account that item 41 (“I feel like vomiting”) should load on both the last two factors) was supported by the data at hand. The goodness-of-fit was evaluated using the comparative fit index (CFI), the Tucker–Lewis index (TLI), and the root-mean-square error of approximation (RMSEA), with its 90% confidence interval (CI). We used the following criteria for model fit [54]: TLI and CFI: values ≥ 0.90 indicated acceptable fit, values ≥ 0.95 indicated excellent fit; RMSEA: values ≤ 0.08 indicated acceptable fit, values ≤ 0.06 indicated excellent fit. Missing values were handled by the full information method implemented in Mplus 7 [55], with which we performed the analyses.

In case of inadequate fit of this model, since we would have to investigate the most suitable measurement model for the Italian BPQ-SF without the support of prior knowledge, we decided on a cross-validation approach, i.e., performing an exploratory factor analysis (EFA) on a random split of the sample in order to find a factor structure that could meet the requirements of a simple approximate structure [56], i.e., each item should substantively (>|0.32|; [57]) load on one factor, while negligibly loading on the others), and a CFA on the other random split.

Before performing these analyses, however, we searched for redundancies and items with low squared multiple correlations (SMC) using the total dataset. The former searches for pairs of items whose intercorrelation is too strong. In factor analysis, these items are likely to yield the so-called “bloated specifics” [58], p. 288), i.e., factors of little substantive interest that result from very highly correlated items that usually share very similar content and/or wording. We considered as redundant items those whose intercorrelation was larger than |0.707| (i.e., more than 50% of shared variance). We then computed SMC for all the remaining items. SMC is the proportion of variance shared by an item with all the others, and it is routinely used by EFA software as an estimate of initial communality, i.e., an estimate of the proportion of variance of an item accounted for by the common factors. Items with SMC smaller than 0.10 are unlikely to contribute substantially to the measurement model and can be removed from the item pool [57].

In order to perform EFA on the first random subsample, we first investigate the optimal number of factors to be extracted through dimensionality analyses, i.e., the scree test [59], the parallel analysis (PA, [60]), and computed the minimum average partial (MAP) correlation statistic [61]. The scree test [59] suggests that the optimal number of factors corresponds to the factors before, which the downward curve of the eigenvalues seems to flatten out. Parallel analysis [60] compares the observed eigenvalues to the eigenvalues generated from a simulated matrix of random data of the same size. Based on the recommendations of Buja & Eyuboglu [62], we performed PA on 1000 random correlation matrices obtained through permutation of the raw data, and following Longman and colleagues [63], we considered the 95th percentile random-generated eigenvalues as the threshold values. Velicer [61] proposed that the optimal number of factors is the one at which the average partial correlation of the variables (i.e., the MAP statistic) reaches its minimum after partialling out the factors.

Once determined the optimal number of factors, we could perform the exploratory analyses, always on the first random subsample. We used exploratory structural equation modeling (ESEM, [64]) with WLSMV estimation, theta parameterization, and GEOMIN rotation. ESEM allows for the estimation of all factor loadings (subject to the constraints necessary for identification) and, in general, for an exploration of complex factor structures (similarly to EFA) while allowing access to parameter estimates, standard errors, goodness-of-fit (GOF) statistics, and modeling flexibility (e.g., correlating error variances, obtaining factor scores corrected for measurement error, etc.)—all features that are otherwise commonly associated with CFA. The choice of the final model relied on the GOF indices (using the same criteria described above for the CFA) and the best approximation of a simple structure. As ESEM allows the estimation of the standard errors of loadings, we considered as substantial those loadings whose 95% confidence interval was entirely over the |0.32| threshold.

Once determined a measurement model through ESEM, we used the data from the other random subsample to test its fit using CFA. Together with the obtained factor model, we also tested alternative models. Two parsimonious models, such as a single factor model and an independent-factor model, and a bifactor model, i.e., a model where the items loaded on general body awareness and reactivity factor, and on specific factors, allowed us to examine the reliability of the total score of the BPQ-22. Besides Cronbach’s alpha, we computed the indices suggested by Rodriguez and colleagues [65] to test whether the single factor score could be considered as sufficiently reliable to be used along with subscale scores. We thus calculated the omega hierarchical coefficient, the explained common variance (ECV), the proportion of items with a relative bias (i.e., the absolute difference between an item’s loading in the unidimensional solution and its general factor loading in the bifactor model, divided by the general factor loading), and the percentage of uncontaminated correlations (PUC, i.e., the number of correlations between items from different group factors divided by the total number of correlations). Support for the use of the total score despite the presence of a multidimensional factor structure is advised if a threshold of 0.80 for the omega hierarchical, and 0.70 for the ECV and the PUC is met [66], and if the proportion of items with a relative bias does not exceed 15% [67].

Construct validity was investigated by computing Spearman correlation coefficients among the observed scores of the BPQ-22 and the other measures administered to the first sample of participants.

The association of the BPQ-22 scores with background variables was tested by specifying a general linear model that included as predictors sex, age, years of education, relationship status, and occupational status. Dunn’s post hoc comparisons with adjustment for false discovery rate were used to test differences between groups in significant categorical predictors.

Finally, we tested the test–retest reliability of the scales on the second sample of participants. The retest coefficient was computed as the Spearman correlation of observed scores at time 1 and time 2, while the stability of scores was evaluated through a Wilcoxon signed-rank test. In order to find evidence for adequate stability of scores, we expected retest coefficients larger than 0.70 (i.e., at least 50% of shared variance) and negligible or low effect sizes (i.e., *r* < 0.30) for the Wilcoxon signed-rank test.

Wherever possible, we computed and reported 95% confidence intervals and measures of effect size. For the data analyses we used IBM^®^ SPSS^®^ 27 software.

## 3. Results

**Figure 1:**
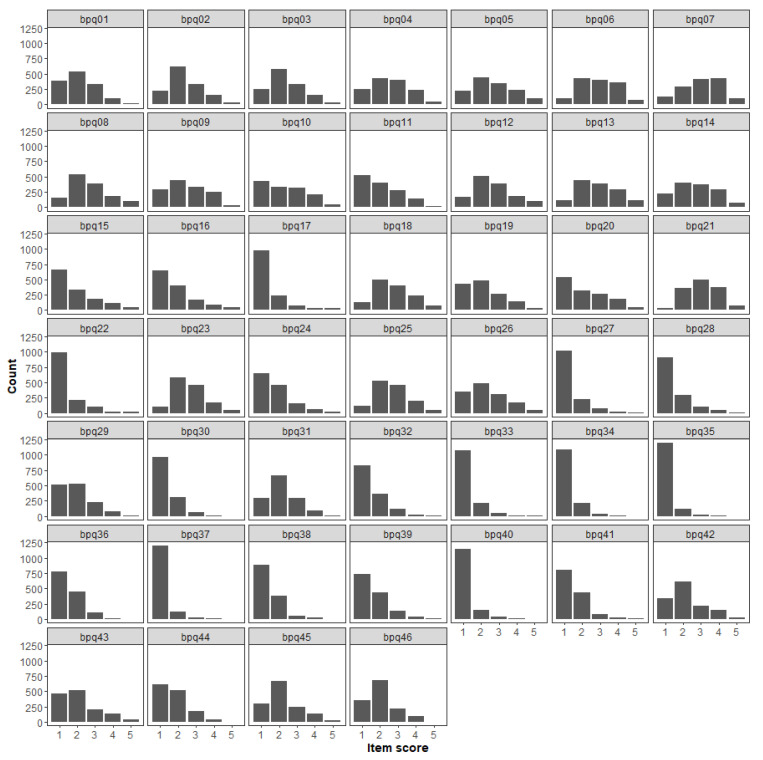
Item raw scores distribution in the total sample (*n* = 1361).

The percentage of missing answers never exceeded 1% (see Table S1 in the Supplementary Materials (SM)), but for some items, the distribution was highly positively skewed (Figure 1), as the 4 and 5 answer options were never or very rarely endorsed. Since we planned to analyze these data as ordinal, the resulting sparseness of the contingency tables of item scores would have affected the estimate of correlation coefficients. We could have removed these items from the item pool, but this would have affected the content validity of the questionnaire (i.e., “the degree to which elements of an assessment instrument are relevant to and representative of the targeted construct for a particular assessment purpose”; [68], p. 238). Hence, we decide to address the issue of rarely endorsed response categories by collapsing categories 3, 4, and 5. This practice has traditionally received mixed consideration in the literature, as there have been supporters (e.g., [69]) and opposers (e.g., [70]). However, a recent simulation study showed that collapsing rarely used response options had negligible effects in establishing valid psychiatric symptom structures, and thus it is a feasible option as long as item scores are specified as ordinal and the sample size is adequate [71], as it is the case of this study. Reducing the response scale from 5 to 3 options ensured that the more severe symptoms states were represented by at least 1% of the sample ([71]; see Table S1 in the SM).

The results of the CFA performed on the total sample to test the adequacy of the original three-factor measurement model revealed a poor fit of this model (χ^2^(985) = 6918.283, *p* < 0.001; comparative fit index (CFI) = 0.789, Tucker–Lewis index (TLI) = 0.778, root mean square error of approximation (RMSEA) = 0.067 (90% confidence interval: 0.065−0.068)). The inspection of modification indices suggested the specification of both cross-loadings (i.e., loadings of some items on the non-expected factor) and correlated uniquenesses (i.e., a correlation between the model-estimated residual variance of a pair of items). We chose to avoid post-hoc modifications of the model since this practice is known to lead to the specification of models that capitalize on chance characteristics of the data and thus are likely to have poor replicability (see, e.g., [72]).

Before using the cross-validation approach described earlier to find the most suitable measurement model for the BPQ items, we searched for redundancies and items with low squared multiple correlations (SMC) using the total dataset. The only pair of items that exceeded the threshold for redundancy was the one that comprised items 26 (“feeling constipated”) and 43 (“I am constipated”). Given that these two items tap into the same symptom, we kept in the item pool only item 43. We then computed SMC for all the remaining items. The only item with an SMC smaller than the threshold was item 11 (“muscle tension in my face”, SMC = 0.058), and was removed from the item pool.

The total sample was then randomly split, and dimensionality and EFA were carried out on the first random subsample (*n* = 668) to determine the optimal number of factors and the most suitable measurement model for the BPQ-SF items. We then tested the fit of this and alternative measurement models using CFA on the other random split (*n* = 693).

**Figure 2:**
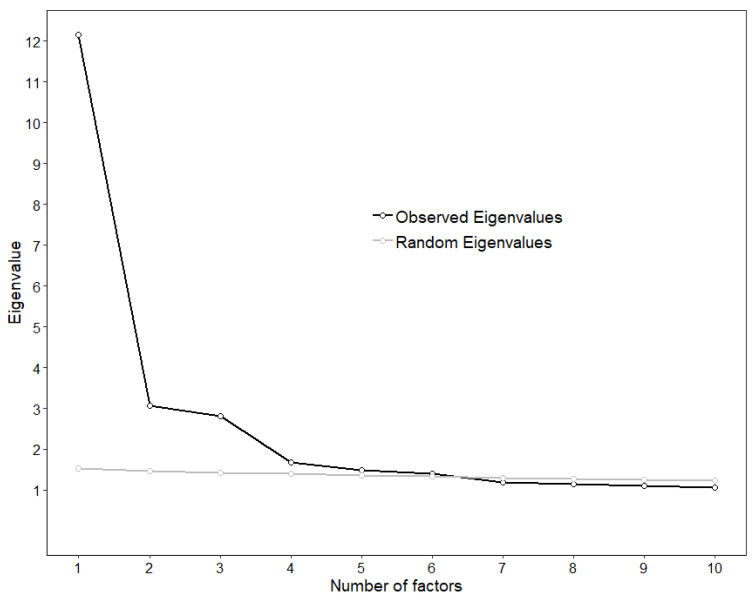
Results of the dimensionality analyses on the first random split of the total sample (*n* = 668).

The line of the scree plot seemed to flatten out at the fourth or fifth factor, suggesting the extraction of three or four factors, respectively (Figure 2). However, the parallel analysis revealed that six observed eigenvalues were larger than the 95th percentile of the corresponding random eigenvalues, while the MAP statistic reached its minimum at the fourth factor (0.0175, 0.0144, 0.0111, 0.0110, 0.0112, 0.0114, 0.0121, 0.0127). Hence, it emerged that the optimal number of factors could be three, four, or six, which would account for 40.98%, 44.78%, and 51.36% of the variance, respectively.

We then used ESEM to test the fit of these models. The results are reported in Table 1, Table 2 and Table 3. The three-factor solution had an acceptable fit (χ^2^ (817) = 1496.322, *p* < 0.001; CFI = 0.942, TLI = 0.933, RMSEA = 0.035 (0.032; 0.038)) and emerged as the most suitable measurement model since we could find at least eight items per factor that had a single loading with a confidence interval entirely over 0.32.

The 4- and 6-factor ESEM models showed a slightly higher fit (χ^2^ (776) = 1279.736, *p* < 0.001; CFI = 0.957, TLI = 0.948, RMSEA = 0.031 [0.028; 0.034] and χ^2^ (697) = 966.611, *p* < 0.001; CFI = 0.977, TLI = 0.969, RMSEA = 0.024 [0.020; 0.028], respectively). However, in the former a factor was defined only by two items that referred to cough issues (2 and 34), while the latter no item showed a single loading with a confidence interval entirely over 0.32 in the second factor. Although none of the dimensionality analyses suggested doing so, we also tested a 5-factor model. This model had an acceptable fit (χ^2^ (736) = 1094.002, *p* < 0.001; CFI = 0.969, TLI = 0.961, RMSEA = 0.027 (0.024; 0.030)), but it did not show evidence of approximated simple structure, as there were three items with loadings whose confidence interval was entirely over 0.32 on two factors, and there were two factors on which loaded only two items (see Table S2 in the SM).

In the final model, the first factor (BPQ-BOA) was loaded by body awareness items (15, 18, 16, 17, 12, 19, 5) and the third factor (BPQ-SUP) by supradiaphragmatic reactivity items (32, 27, 33, 28, 39, 37, 38, 34). The second factor (BPQ-BOA/SUB) comprised some items from the body awareness scale and some from the subdiaphragmatic reactivity scale (45, 42, 14, 44, 13, 46, 7), but all tapping into bloating and digestive issues. Together, these items are comprised in the BPQ-22.

Once determined a measurement model through ESEM, we used the data from the other random subsample to test the fit of these models and of three alternative models (single-factor model, three-independent-factor model, and bifactor model) using CFA. The results are reported in Table 4 and Table 5 and show that both the three-correlated-factor and the bifactor model have an adequate fit. Although the single factor score showed a Cronbach’s alpha of 0.91, the omega hierarchical coefficient was 0.76 (hence lower the recommended threshold of 0.80), and the explained common variance (ECV) was 0.54, suggesting a weak general factor. Moreover, the proportion of items with a relative bias larger than the recommended 15% was 0.46, and the percentage of uncontaminated correlations was 0.69. As none of these indices met the criteria described in the Data Analysis section, we concluded that these results did not support the use of a total score.

Table 6 shows the correlations of the scores on the BPQ-22 scales with the other scales in this study. Given the very large sample size, we refrained from reporting significance levels, as a correlation as low as 0.089 would have been significant at *p* < 0.001 and comment only on effect sizes. The three BPQ-22 scales showed very similarly moderate (i.e., 0.30 < ρ < 0.50) correlations with the SSAS, suggesting that higher scores on any scale are associated with a higher tendency to experience somatic and visceral sensations as intense and disturbing. The body awareness scale scores tended to be less correlated with the other measures than the other two scales of the BPQ-22, as coefficients were always in the weak range (i.e., 0.10 < ρ < 0.30). Despite not being much correlated with one another, the BPQ-SUP and the BPQ-BOA/SUB showed a similar pattern of moderate, positive correlations with the DASS scales, suggesting that higher scores tend to be associated with higher levels of anxiety, depression, and stress symptoms. The same two BPQ-22 scales had a strong correlation (i.e., ρ > 0.50) with MSPQ, which is consistent with the expectation that individuals with higher levels of supra- and subdiaphragmatic reactivity tend to report higher levels of somatic complaints, supporting the convergent construct validity of the scales. Finally, the BPQ-SUP scale had low-to-moderate correlations with the OBQ scales, while the other two BPQ-22 scales showed only weak correlations with them. This result indicates that individuals with a higher supradiaphragmatic reactivity tend to generally report more intense misappraisals of intrusive thoughts. Taken together, these results seem to support the convergent and discriminant validity of the BPQ-22 scales.

We then tested the association of the BPQ-22 scores with background variables. For the BOA/SUB scale we found that females scored higher than males (*F*(1, 1319) = 21.59, *p* < 0.001; estimated marginal means (EMMs): females: 16.12 (standard error (se) = 0.52); males: 14.54 (SE = 0.59); *d* = 0.33 (0.19; 0.46)), that the scores decreased with age (*F*(1, 1319) = 8.00, *p* = 0.005; *r* = −0.08 (−0.13; −0.02)), and that there were differences across relationship status categories (*F*(1, 1319) = 3.37, *p* = 0.018; η^2^ = 0.09 (0.01; 0.13)). However, when we performed post hoc comparisons between the categories of this variable, none was statistically significant after the Dunn adjustment for false discovery rate for a family of 4 estimates (see SM for details on EMMs and contrasts). For the SUP scale we found that females scored higher than males (*F*(1, 1330) = 12.26, *p* < 0.001; estimated marginal means (EMMs): females: 11.10 (SE = 0.37); males: 10.17 (SE = 0.42); d = 0.24 (0.11; 0.38)), that the scores decreased with years of education (*F*(1, 1319) = 5.07, *p* = 0.024; *r* = −0.06 (−0.11; −0.01)), and that there were differences across relationship status categories (*F*(1, 1319) = 3.61, *p* = 0.013; η^2^ = 0.09 (0.02; 0.14)). Dunn-adjusted post hoc comparisons revealed that single participants tended to endorse higher scores than participants in a relationship (see Tables S3–S6 in SM for details on EMMs and contrasts). For the BOA scale, we only found that scores tended to decrease with age (*F*(1, 1319) = 31.60, *p* < 0.001; r = −0.15 (−0.20; −0.10)). Beyond statistical significance, the size of the effects suggests that the association of the BPQ-22 scores with background variables was generally weak.

Finally, we tested the test–retest reliability of the scales on the second sample of participants. The results are reported in Table 7 and show that the scores were fairly consistent in a 3 week period, as all test–retest Spearman correlations were well above 0.70. We also carried out the intraclass correlation coefficient (ICC) that confirmed the consistency of the scores in a 3 week period (BOA/SUB: ICC = 0.869, (0.80; 0.91), *p* < 0.0001; SUP: ICC = 0.875, (0.81; 0.91), *p* < 0.0001; BOA: ICC = 0.901, (0.85; 0.93), *p* < 0.0001). In addition, we carried out the Wilcoxon signed-rank test to investigate the absolute stability of scores, and found that, despite the tests were statistically or marginally significant, the effect sizes suggested that these differences were negligible (BOA/SUB: Z(98) = −1.95, *p* = 0.05, r = −0.14; SUP: Z(98) = −2.12, *p* = 0.034, r = −0.15; BOA: Z(98) = −3.12, *p* = 0.002, r = −0.22).

## 4. Discussion

The aim of the present study was to validate the Italian version of the BPQ-SF [36], evaluating the possibility of collapsing response categories, item reduction, its factor structure, reliability, convergent and discriminant validity. Our results supported the collapsing of response categories 3, 4, 5, the item reduction from 46 to 22, a three-factor structure (consisting of a body awareness factor, a supradiaphragmatic factor and a subdiaphragmatic/body awareness factor), the test–retest reliability and the convergent and discriminant validity of the scale.

Though collapsing response categories have been both supported (e.g., [69]) and opposed (e.g., [70]), to preserve the content validity of the questionnaire, we collapsed response categories 3, 4, 5. Accordingly, a recent simulation study showed that collapsing infrequently used response options had minor effects in establishing valid psychiatric symptom structures. Therefore, it represents a viable option as long as item scores are specified as ordinal and the sample size is adequate [71]. Hence, the item scores and the sample size of this study were adequate to undergo this kind of analysis.

The three-factor structure is in line with the original findings by Cabrera et al. [36] and with the findings by Wang et. al. [73] for the Chinese validation of BPQ-SF. The BOA factor, consisting of items related to the upper parts of the body (e.g., “watering or tearing of my eyes”) or to the whole body (e.g., “goose-bumps”) may reflect the convergence of cranial and spinal pathways integration in the brainstem, while the SUP factor may reflect the function exerted by VVC whose fibers originate in the NA in the brainstem. Interestingly, we found that the third factor, BOA/SUB, included three items from the original BOA subscale as well as four items that are related to subdiaphragmatic issues, all tapping into bloating and digestive issues. Though it may seem surprising or unexpected, there is evidence that may account for this finding. Recently, Kaelberer et. al. [74], using optogenetics and whole-cell patch–clamp recordings, found remarkable evidence for a gut–brain neural circuit involved in nutrient sensory transduction through enteroendocrine cells. Nevertheless, unlike other sensory epithelial cells, no synaptic connection has been established between enteroendocrine cells and a cranial nerve [75]. It is believed that these cells can act on nerves only through an indirect effect mediated by the slow endocrine action of hormones, like cholecystokinin. However, circulating concentrations of cholecystokinin typically reach their highest levels just several minutes after food ingestion. Hence, this evidence suggests that the central nervous system may sense gut sensory information through faster synaptic transmission. A monosynaptic tracing, using rabies virus, allowed us to discover that enteroendocrine cells project to vagal nodose neurons in one synapse. Furthermore, optogenetic stimulation of enteroendocrine cells generated excitatory postsynaptic potentials in synaptically-associated nodose neurons within milliseconds. Eventually, cholecystokinin and glutamate pharmacological inactivation experiments showed that enteroendocrine cells (termed neuropod cells; [74]) use glutamate as a neurotransmitter to relay sensory gut information to the brainstem. Remarkably, vagal nodose neurons are pseudounipolar afferent neurons so that the information is relayed in just one synapse to the brainstem and, in particular, to the nucleus of the tractus solitarius (NTS). NTS is a brainstem nucleus that represents an integrative hub for olfactory and gustatory information [76] that is upstream to both the NA and the DMNX. In addition to receiving sensory information also from the gut, NTS receives information also from the insula [77] that is typically believed to play a critical role in human interoceptive awareness [78]. Thus, the NTS seems to integrate subdiaphragmatic reactivity information originating directly from the gut (e.g., the information outflow originating from neuropod cells) and bodily awareness information originating from the insula. Accordingly, the SUB component of our BOA/SUB factor may be represented by gut sensory information projected by cells like neuropod cells and relayed by NTS to cardioinhibitory fibers stemming from DMNX, while the BOA component may be represented by bodily awareness information projected by insula and relayed by NTS to cardioinhibitory fibers stemming from NA. Together, this may represent the implementation of the immobilization without fear state, through a neuroception of safety [29], that, in the polyvagal theory, is believed to require a co-activation of the NA and the DMNX fibers. The co-activation of myelinated NA fibers would assure a sense of safety given by the awareness of one’s own bodily state, that could be, or promote, a portal to self-compassion [79,80,81,82,83,84,85,86,87,88,89,90].

Regarding convergent validity, the three BPQ-22 scales showed moderate correlations with the SSAS, suggesting that higher scores on any scale are associated with a higher tendency to experience somatic and visceral sensations as intense and disturbing. The BPQ-SUP and the BPQ-BOA/SUB showed a similar pattern of moderate, positive correlations with the DASS-21 subscales, suggesting that higher scores tend to be associated with higher anxiety levels, depression, and distress symptoms. Accordingly, anxiety, depression and distress symptom appraisals tend to be highly rooted in bodily perception. Furthermore, BPQ-SUP and BPQ-BOA/SUB scales strongly correlated with MSPQ, which suggests that individuals with higher levels of supra- and subdiaphragmatic reactivity tend to experience higher levels of somatic complaints, supporting the convergent construct validity of the scales. Finally, the BPQ-SUP scale had low-to-moderate correlations with the OBQ scales, while the other two BPQ-22 scales showed only weak correlations with them. This result indicates that individuals with a higher supradiaphragmatic reactivity tend to generally report more intense misappraisals of intrusive thoughts. Overall, these results seem to support the convergent and discriminant validity of the BPQ-22 scales.

Regarding background demographic variables, we found that single participants tended to endorse higher scores in the SUP than participants in a relationship. These results may be explained considering that, according to the polyvagal theory, intimacy and romantic relationships require immobilization without fear, which is implemented by a co-activation of the VVC and the DVC, thus implying the whole parasympathetic nervous system. VVC activation, requiring the activity of the myelinated fibers of the NA, may contribute to homeostatically regulate sympathetic and supradiaphragmatic activation. Single and lonely people may present a state, or trait, tendency to rely on the sympathetic nervous system [91,92], associated with a tendency not to trust the parasympathetic nervous system states that may be sensed as unsafe. For the BOA scale, we show that scores tended to decrease with age, a finding that is in line with previous research demonstrating that aging tends to increase sensory thresholds for a variety of exteroceptive and proprioceptive stimuli [93]. Unsurprisingly, age-related declines have been demonstrated in VVC cardiac autonomic regulation through the assessment of respiratory sinus arrhythmia [94,95,96]. These results suggest that the co-occurring bodily sensations changes may reflect dampened sensory transmission between body and brain over time.

Regarding the test–retest reliability of the scales, we evaluated it on another sample of participants. The scores were consistent after a 3 week period, as all test–retest correlations were well above 0.70, showing that BPQ-22 has a good test–retest reliability.

This study has some limitations that should be outlined. First, the questionnaire’s psychometric properties were evaluated only in a large non-clinical sample recruited from the general Italian population; further studies should confirm its three-factor structure and adequate reliability and validity in clinical samples, although this would require large clinical sample sizes. Second, though the scale had a sound pattern of convergent and discriminant validity when the present study was planned, other tools concerning a neurophysiologically informed body perception assessment were not available. Thus, future studies using other neurophysiologically informed convergent measures would confirm our conclusions.

## Figures and Tables

**Table 1:** Loading matrix and factor correlations of the three-factor exploratory structural equation modeling solution.

**Item**	**F1**	**F2**	**F3**
BPQ01	0.33 [0.21; 0.45]	−0.12 [−0.25; 0.02]	0.32 [0.19; 0.46]
BPQ02	0.33 [0.22; 0.45]	−0.07 [−0.20; 0.07]	0.27 [0.13; 0.41]
BPQ03	0.41 [0.31; 0.52]	0.13 [−0.01; 0.27]	0.21 [0.06; 0.35]
BPQ04	**0.46 [0.36; 0.57]**	0.09 [−0.05; 0.23]	0.12 [−0.03; 0.27]
BPQ05	**0.59 [0.49; 0.69]**	0.02 [−0.11; 0.15]	−0.06 [−0.19; 0.08]
BPQ06	0.39 [0.26; 0.52]	0.30 [0.16; 0.43]	−0.08 [−0.20; 0.05]
BPQ07	0.08 [−0.11; 0.27]	**0.63 [0.49; 0.76]**	−0.10 [−0.24; 0.04]
BPQ08	0.39 [0.27; 0.50]	0.23 [0.10; 0.36]	0.00 [−0.09; 0.10]
BPQ09	0.37 [0.24; 0.49]	0.31 [0.19; 0.44]	0.05 [−0.08; 0.18]
BPQ10	0.03 [−0.11; 0.17]	**0.45 [0.33; 0.56]**	0.00 [−0.12; 0.13]
BPQ12	**0.54 [0.42; 0.65]**	0.20 [0.06; 0.33]	−0.12 [−0.26; 0.01]
BPQ13	0.15 [−0.07; 0.36]	**0.76 [0.63; 0.89]**	−0.11 [−0.23; 0.02]
BPQ14	0.02 [−0.16; 0.20]	**0.76 [0.63; 0.89]**	−0.05 [−0.17; 0.07]
BPQ15	**0.61 [0.51; 0.70]**	0.03 [−0.10; 0.15]	0.01 [−0.11; 0.12]
BPQ16	**0.49 [0.38; 0.59]**	−0.02 [−0.14; 0.09]	0.02 [−0.11; 0.14]
BPQ17	**0.68 [0.57; 0.80]**	0.00 [−0.05; 0.05]	0.16 [−0.01; 0.33]
BPQ18	**0.49 [0.38; 0.60]**	0.11 [−0.02; 0.24]	−0.08 [−0.21; 0.04]
BPQ19	**0.55 [0.46; 0.65]**	0.02 [−0.09; 0.12]	0.04 [−0.11; 0.18]
BPQ20	0.26 [0.15; 0.37]	0.16 [0.03; 0.29]	0.12 [−0.01; 0.26]
BPQ21	0.30 [0.14; 0.45]	0.42 [0.28; 0.55]	0.03 [−0.10; 0.16]
BPQ22	0.44 [0.30; 0.57]	0.22 [0.07; 0.36]	−0.02 [−0.13; 0.10]
BPQ23	0.41 [0.28; 0.53]	0.31 [0.18; 0.44]	0.05 [−0.08; 0.19]
BPQ24	**0.48 [0.36; 0.60]**	−0.08 [−0.23; 0.07]	0.40 [0.25; 0.55]
BPQ25	**0.51 [0.39; 0.63]**	0.24 [0.09; 0.39]	0.16 [0.01; 0.32]
BPQ27	−0.10 [−0.23; 0.03]	0.26 [0.03; 0.48]	**0.55 [0.39; 0.71]**
BPQ28	0.12 [−0.02; 0.26]	0.03 [−0.14; 0.19]	**0.51 [0.37; 0.65]**
BPQ29	0.18 [0.06; 0.29]	0.21 [0.06; 0.37]	0.29 [0.15; 0.43]
BPQ30	0.03 [−0.08; 0.13]	0.10 [−0.13; 0.32]	**0.60 [0.46; 0.75]**
BPQ31	0.09 [−0.03; 0.20]	0.24 [0.04; 0.44]	0.45 [0.30; 0.60]
BPQ32	0.02 [−0.07; 0.11]	0.19 [−0.05; 0.42]	**0.62 [0.47; 0.77]**
BPQ33	−0.10 [−0.24; 0.05]	0.13 [−0.15; 0.42]	**0.77 [0.59; 0.95]**
BPQ34	0.04 [−0.12; 0.20]	−0.08 [−0.29; 0.13]	**0.56 [0.40; 0.71]**
BPQ35	0.02 [−0.10; 0.13]	−0.09 [−0.35; 0.18]	**0.78 [0.61; 0.94]**
BPQ36	0.04 [−0.08; 0.15]	0.26 [0.09; 0.44]	0.35 [0.21; 0.49]
BPQ37	−0.13 [−0.30; 0.04]	0.03 [−0.19; 0.26]	**0.73 [0.56; 0.90]**
BPQ38	0.13 [0.00; 0.27]	−0.03 [−0.22; 0.15]	**0.59 [0.45; 0.73]**
BPQ39	−0.03 [−0.13; 0.07]	0.20 [−0.01; 0.42]	**0.57 [0.42; 0.72]**
BPQ40	0.16 [0.02; 0.31]	0.09 [−0.09; 0.26]	0.30 [0.15; 0.46]
BPQ41	0.09 [−0.05; 0.22]	0.36 [0.21; 0.50]	0.17 [0.04; 0.30]
BPQ42	−0.14 [−0.35; 0.07]	**0.72 [0.61; 0.84]**	0.00 [−0.08; 0.08]
BPQ43	−0.03 [−0.14; 0.08]	0.29 [0.16; 0.43]	0.14 [0.00; 0.27]
BPQ44	−0.25 [−0.46; −0.04]	**0.78 [0.65; 0.91]**	0.06 [−0.04; 0.16]
BPQ45	−0.28 [−0.53; −0.02]	**0.93 [0.81; 1.05]**	0.00 [−0.04; 0.04]
BPQ46	−0.02 [−0.14; 0.10]	**0.44 [0.32; 0.56]**	0.07 [−0.05; 0.18]
ρ with F2	0.43 [0.21; 0.64]		
ρ with F3	0.37 [0.19; 0.54]	0.44 [0.22; 0.67]	

Note: Bracketed values and the 95% confidence interval of the loading estimate. Bold values indicate that this interval is entirely over |0.32| (*n* = 668).

**Table 2:** Loading matrix and factor correlations of the four-factor exploratory structural equation modeling solution (data from the first random subsample, *n* = 668).

Item	F1	F2	F3	F4
BPQ01	0.26 [0.10; 0.41]	0.06 [−0.09; 0.22]	−0.04 [−0.14; 0.05]	0.42 [0.29; 0.55]
BPQ02	0.26 [0.09; 0.44]	−0.03 [−0.16; 0.10]	0.03 [−0.06; 0.13]	**0.46 [0.34; 0.59]**
BPQ03	**0.43 [0.32; 0.53]**	0.13 [0.00; 0.25]	0.08 [−0.03; 0.18]	0.21 [0.07; 0.34]
BPQ04	**0.51 [0.43; 0.60]**	0.20 [0.08; 0.32]	−0.06 [−0.16; 0.04]	−0.01 [−0.11; 0.10]
BPQ05	**0.61 [0.52; 0.69]**	−0.02 [−0.13; 0.09]	−0.08 [−0.19; 0.03]	0.02 [−0.10; 0.15]
BPQ06	**0.46 [0.37; 0.55]**	−0.01 [−0.12; 0.09]	0.19 [0.07; 0.31]	−0.02 [−0.13; 0.09]
BPQ07	0.23 [0.09; 0.37]	0.06 [−0.08; 0.19]	**0.49 [0.38; 0.60]**	−0.15 [−0.27; −0.02]
BPQ08	0.41 [0.28; 0.54]	−0.12 [−0.24; 0.01]	0.23 [0.12; 0.34]	0.21 [0.06; 0.36]
BPQ09	**0.45 [0.36; 0.55]**	0.13 [0.01; 0.26]	0.18 [0.07; 0.28]	−0.02 [−0.13; 0.09]
BPQ10	0.14 [0.01; 0.28]	0.15 [0.01; 0.28]	0.33 [0.22; 0.44]	−0.13 [−0.24; −0.02]
BPQ12	**0.61 [0.53; 0.69]**	0.01 [−0.09; 0.11]	0.04 [−0.06; 0.14]	−0.11 [−0.24; 0.03]
BPQ13	0.30 [0.18; 0.42]	−0.04 [−0.12; 0.05]	**0.66 [0.57; 0.74]**	−0.02 [−0.09; 0.06]
BPQ14	0.17 [0.05; 0.30]	0.01 [−0.08; 0.09]	**0.67 [0.58; 0.76]**	−0.01 [−0.08; 0.07]
BPQ15	**0.61 [0.51; 0.71]**	−0.05 [−0.15; 0.05]	−0.03 [−0.13; 0.07]	0.15 [−0.02; 0.31]
BPQ16	0.45 [0.31; 0.60]	−0.16 [−0.29; −0.02]	0.01 [−0.08; 0.10]	0.27 [0.13; 0.42]
BPQ17	**0.68 [0.55; 0.81]**	0.09 [−0.04; 0.22]	−0.08 [−0.18; 0.02]	0.22 [0.03; 0.40]
BPQ18	**0.50 [0.39; 0.62]**	−0.14 [−0.26; −0.02]	0.08 [−0.02; 0.18]	0.12 [−0.04; 0.29]
BPQ19	**0.54 [0.43; 0.65]**	−0.06 [−0.17; 0.06]	−0.01 [−0.10; 0.08]	0.19 [0.04; 0.34]
BPQ20	0.30 [0.20; 0.40]	0.14 [0.01; 0.26]	0.08 [−0.03; 0.19]	0.06 [−0.05; 0.17]
BPQ21	0.41 [0.27; 0.56]	0.21 [0.06; 0.36]	0.23 [0.11; 0.36]	−0.15 [−0.29; −0.01]
BPQ22	**0.48 [0.35; 0.60]**	−0.03 [−0.16; 0.10]	0.15 [0.02; 0.29]	0.09 [−0.06; 0.24]
BPQ23	**0.50 [0.40; 0.60]**	0.18 [0.06; 0.31]	0.14 [0.03; 0.25]	−0.07 [−0.19; 0.05]
BPQ24	0.42 [0.26; 0.58]	0.14 [−0.04; 0.32]	−0.04 [−0.13; 0.04]	0.46 [0.31; 0.61]
BPQ25	**0.62 [0.51; 0.72]**	0.36 [0.23; 0.48]	−0.01 [−0.07; 0.06]	−0.12 [−0.26; 0.02]
BPQ27	−0.01 [−0.10; 0.08]	**0.65 [0.55; 0.74]**	0.10 [−0.01; 0.21]	−0.01 [−0.13; 0.11]
BPQ28	0.13 [0.02; 0.24]	**0.46 [0.33; 0.59]**	−0.05 [−0.16; 0.07]	0.19 [0.03; 0.34]
BPQ29	0.26 [0.14; 0.38]	**0.47 [0.36; 0.57]**	0.02 [−0.07; 0.10]	−0.10 [−0.23; 0.03]
BPQ30	0.02 [−0.07; 0.11]	0.45 [0.27; 0.64]	0.08 [−0.03; 0.20]	0.33 [0.17; 0.49]
BPQ31	0.17 [0.05; 0.28]	**0.55 [0.45; 0.65]**	0.08 [−0.02; 0.18]	0.00 [−0.11; 0.11]
BPQ32	0.10 [−0.01; 0.21]	**0.70 [0.61; 0.80]**	0.01 [−0.08; 0.10]	0.03 [−0.10; 0.16]
BPQ33	−0.04 [−0.11; 0.04]	**0.80 [0.68; 0.92]**	−0.01 [−0.11; 0.09]	0.12 [−0.07; 0.30]
BPQ34	−0.06 [−0.20; 0.08]	0.22 [−0.03; 0.46]	0.05 [−0.07; 0.17]	**0.55 [0.39; 0.70]**
BPQ35	−0.05 [−0.14; 0.05]	0.52 [0.29; 0.75]	−0.04 [−0.15; 0.06]	0.47 [0.28; 0.67]
BPQ36	0.08 [−0.02; 0.19]	0.33 [0.20; 0.45]	0.20 [0.09; 0.31]	0.13 [0.00; 0.27]
BPQ37	−0.14 [−0.27; −0.01]	**0.59 [0.39; 0.78]**	0.02 [−0.08; 0.13]	0.32 [0.10; 0.53]
BPQ38	0.07 [−0.05; 0.20]	0.34 [0.15; 0.54]	0.02 [−0.07; 0.11]	0.45 [0.31; 0.60]
BPQ39	0.04 [−0.06; 0.13]	**0.62 [0.52; 0.73]**	0.07 [−0.03; 0.17]	0.07 [−0.07; 0.21]
BPQ40	0.14 [−0.01; 0.30]	0.13 [−0.05; 0.32]	0.11 [−0.03; 0.26]	0.31 [0.14; 0.47]
BPQ41	0.13 [0.01; 0.24]	0.07 [−0.07; 0.20]	0.36 [0.24; 0.47]	0.21 [0.09; 0.33]
BPQ42	−0.03 [−0.09; 0.03]	−0.06 [−0.16; 0.04]	**0.73 [0.65; 0.80]**	0.12 [0.03; 0.22]
BPQ43	0.04 [−0.07; 0.15]	0.21 [0.10; 0.33]	0.21 [0.10; 0.32]	−0.04 [−0.16; 0.08]
BPQ44	−0.13 [−0.23; −0.03]	0.02 [−0.06; 0.10]	**0.77 [0.69; 0.85]**	0.10 [0.00; 0.21]
BPQ45	−0.09 [−0.20; 0.02]	0.06 [−0.01; 0.14]	**0.85 [0.78; 0.92]**	−0.02 [−0.10; 0.05]
BPQ46	0.06 [−0.04; 0.16]	0.06 [−0.05; 0.17]	0.41 [0.31; 0.51]	0.06 [−0.04; 0.17]
ρ with F2	0.36 [0.22; 0.49]			
ρ with F3	0.35 [0.26; 0.44]	0.45 [0.36; 0.53]		
ρ with F4	0.16 [0.02; 0.31]	0.27 [0.12; 0.43]	0.10 [−0.05; 0.25]	

Note: Bracketed values and the 95% confidence interval of the loading estimate. Bold values indicate that this interval is entirely over |0.32.

**Table 3:** Loading matrix and factor correlations of the six-factor exploratory structural equation modeling solution (data from the first random subsample, *n* = 668).

Item	F1	F2	F3	F4	F5	F6
BPQ01	0.03 [−0.08; 0.15]	0.21 [−0.08; 0.49]	**0.58 [0.43; 0.72]**	0.05 [−0.06; 0.16]	−0.07 [−0.17; 0.04]	−0.14 [−0.28; 0.00]
BPQ02	0.06 [−0.08; 0.20]	0.12 [−0.18; 0.41]	**0.61 [0.47; 0.76]**	−0.08 [−0.20; 0.05]	0.03 [−0.05; 0.11]	−0.11 [−0.22; 0.01]
BPQ03	0.21 [0.02; 0.40]	0.26 [0.00; 0.52]	0.34 [0.17; 0.51]	0.06 [−0.07; 0.19]	0.04 [−0.07; 0.14]	0.04 [−0.08; 0.16]
BPQ04	0.41 [0.21; 0.60]	0.18 [−0.06; 0.41]	0.04 [−0.09; 0.16]	0.16 [0.01; 0.31]	−0.10 [−0.21; 0.02]	0.13 [−0.02; 0.27]
BPQ05	0.45 [0.23; 0.66]	0.16 [−0.07; 0.40]	0.14 [−0.04; 0.32]	−0.12 [−0.27; 0.02]	−0.11 [−0.23; 0.01]	0.14 [0.00; 0.29]
BPQ06	0.44 [0.28; 0.60]	0.15 [−0.12; 0.42]	−0.08 [−0.21; 0.06]	0.09 [−0.06; 0.25]	0.15 [0.01; 0.29]	−0.04 [−0.17; 0.09]
BPQ07	0.02 [−0.12; 0.16]	0.50 [0.30; 0.70]	−0.04 [−0.20; 0.11]	0.09 [−0.10; 0.27]	0.40 [0.19; 0.61]	−0.02 [−0.12; 0.08]
BPQ08	0.35 [0.21; 0.49]	0.07 [−0.20; 0.33]	0.22 [0.07; 0.37]	−0.06 [−0.19; 0.06]	0.22 [0.10; 0.33]	−0.09 [−0.22; 0.04]
BPQ09	0.31 [0.11; 0.50]	0.25 [0.02; 0.48]	0.05 [−0.08; 0.19]	0.09 [−0.05; 0.22]	0.13 [0.00; 0.27]	0.11 [−0.03; 0.24]
BPQ10	−0.01 [−0.13; 0.12]	0.39 [0.22; 0.56]	−0.07 [−0.22; 0.08]	0.18 [0.00; 0.37]	0.26 [0.08; 0.44]	−0.01 [−0.12; 0.09]
BPQ12	**0.53 [0.33; 0.73]**	0.10 [−0.11; 0.32]	−0.07 [−0.20; 0.06]	−0.07 [−0.20; 0.07]	0.02 [−0.08; 0.12]	0.20 [0.05; 0.35]
BPQ13	0.17 [0.01; 0.32]	0.23 [−0.01; 0.46]	0.05 [−0.05; 0.15]	−0.10 [−0.22; 0.02]	**0.62 [0.48; 0.76]**	0.10 [−0.02; 0.22]
BPQ14	0.03 [−0.08; 0.14]	0.34 [0.11; 0.56]	0.04 [−0.05; 0.14]	0.06 [−0.07; 0.18]	**0.63 [0.46; 0.79]**	−0.07 [−0.18; 0.04]
BPQ15	**0.77 [0.67; 0.88]**	−0.25 [−0.53; 0.02]	−0.03 [−0.12; 0.07]	0.06 [−0.06; 0.17]	0.00 [−0.08; 0.08]	0.00 [−0.09; 0.08]
BPQ16	**0.65 [0.51; 0.78]**	−0.29 [−0.61; 0.03]	0.09 [−0.05; 0.22]	0.04 [−0.05; 0.13]	0.05 [−0.04; 0.15]	−0.21 [−0.34; −0.07]
BPQ17	**0.60 [0.44; 0.77]**	0.01 [−0.22; 0.25]	0.23 [0.07; 0.38]	0.08 [−0.05; 0.21]	−0.08 [−0.19; 0.02]	0.06 [−0.06; 0.17]
BPQ18	**0.65 [0.53; 0.76]**	−0.17 [−0.46; 0.12]	−0.04 [−0.15; 0.07]	0.02 [−0.08; 0.12]	0.09 [−0.01; 0.20]	−0.10 [−0.23; 0.03]
BPQ19	**0.56 [0.43; 0.70]**	−0.13 [−0.39; 0.14]	0.15 [0.02; 0.28]	−0.04 [−0.15; 0.07]	0.01 [−0.09; 0.10]	0.02 [−0.08; 0.13]
BPQ20	0.19 [0.02; 0.35]	0.15 [−0.03; 0.34]	0.12 [−0.02; 0.27]	0.08 [−0.06; 0.23]	0.06 [−0.06; 0.18]	0.08 [−0.05; 0.21]
BPQ21	0.26 [0.04; 0.48]	0.16 [−0.02; 0.33]	−0.03 [−0.16; 0.10]	−0.02 [−0.13; 0.09]	0.21 [0.08; 0.35]	0.37 [0.23; 0.52]
BPQ22	0.29 [0.05; 0.52]	0.27 [−0.02; 0.55]	0.22 [0.01; 0.42]	−0.08 [−0.25; 0.08]	0.11 [−0.04; 0.27]	0.02 [−0.11; 0.15]
BPQ23	0.32 [0.10; 0.54]	0.20 [−0.01; 0.41]	0.05 [−0.07; 0.18]	0.01 [−0.09; 0.11]	0.11 [−0.02; 0.24]	0.28 [0.14; 0.42]
BPQ24	0.20 [0.02; 0.37]	0.11 [−0.19; 0.41]	**0.61 [0.49; 0.73]**	0.04 [−0.05; 0.14]	−0.06 [−0.15; 0.04]	0.02 [−0.08; 0.12]
BPQ25	0.51 [0.26; 0.76]	−0.03 [−0.13; 0.08]	−0.03 [−0.11; 0.05]	0.02 [−0.05; 0.09]	−0.02 [−0.08; 0.04]	**0.60 [0.46; 0.75]**
BPQ27	0.05 [−0.05; 0.16]	0.15 [−0.13; 0.42]	−0.13 [−0.27; 0.01]	**0.72 [0.58; 0.86]**	0.09 [−0.03; 0.20]	−0.03 [−0.12; 0.07]
BPQ28	0.15 [0.02; 0.28]	0.03 [−0.15; 0.21]	0.11 [−0.03; 0.24]	**0.54 [0.41; 0.67]**	−0.06 [−0.17; 0.05]	−0.04 [−0.14; 0.07]
BPQ29	0.13 [−0.09; 0.34]	−0.02 [−0.12; 0.08]	0.00 [−0.10; 0.11]	0.12 [−0.01; 0.26]	0.01 [−0.06; 0.08]	**0.58 [0.46; 0.70]**
BPQ30	0.00 [−0.11; 0.11]	−0.09 [−0.28; 0.10]	0.29 [0.12; 0.46]	0.41 [0.27; 0.55]	0.09 [−0.03; 0.21]	0.09 [−0.04; 0.22]
BPQ31	0.04 [−0.09; 0.17]	0.02 [−0.07; 0.11]	0.09 [−0.05; 0.22]	0.28 [0.15; 0.41]	0.07 [−0.03; 0.17]	**0.45 [0.34; 0.56]**
BPQ32	0.01 [−0.09; 0.11]	0.18 [−0.02; 0.38]	0.04 [−0.06; 0.13]	**0.64 [0.50; 0.77]**	−0.02 [−0.10; 0.05]	0.18 [0.07; 0.30]
BPQ33	0.04 [−0.06; 0.14]	0.07 [−0.20; 0.35]	−0.02 [−0.10; 0.07]	**0.89 [0.77; 1.01]**	−0.02 [−0.11; 0.06]	−0.05 [−0.15; 0.06]
BPQ34	−0.24 [−0.41; −0.06]	−0.04 [−0.19; 0.11]	**0.69 [0.52; 0.85]**	0.08 [−0.10; 0.26]	0.07 [−0.06; 0.20]	0.02 [−0.08; 0.12]
BPQ35	−0.04 [−0.16; 0.09]	−0.19 [−0.44; 0.06]	0.41 [0.17; 0.66]	0.48 [0.28; 0.68]	−0.02 [−0.12; 0.07]	0.07 [−0.06; 0.19]
BPQ36	−0.03 [−0.13; 0.06]	−0.06 [−0.19; 0.06]	0.23 [0.07; 0.40]	0.06 [−0.08; 0.20]	0.22 [0.10; 0.33]	0.36 [0.23; 0.48]
BPQ37	−0.01 [−0.13; 0.12]	−0.26 [−0.51; −0.01]	0.14 [−0.14; 0.42]	**0.61 [0.44; 0.79]**	0.05 [−0.07; 0.16]	0.06 [−0.08; 0.20]
BPQ38	0.03 [−0.08; 0.14]	−0.08 [−0.29; 0.13]	0.43 [0.28; 0.58]	0.34 [0.20; 0.48]	0.03 [−0.06; 0.12]	−0.03 [−0.12; 0.07]
BPQ39	−0.05 [−0.15; 0.05]	0.00 [−0.10; 0.09]	0.11 [−0.05; 0.26]	0.43 [0.29; 0.56]	0.06 [−0.04; 0.16]	0.35 [0.23; 0.47]
BPQ40	0.09 [−0.07; 0.25]	−0.10 [−0.30; 0.11]	0.33 [0.14; 0.51]	0.05 [−0.11; 0.21]	0.12 [−0.03; 0.27]	0.10 [−0.07; 0.26]
BPQ41	0.06 [−0.06; 0.19]	−0.01 [−0.16; 0.15]	0.24 [0.10; 0.37]	−0.01 [−0.13; 0.12]	0.36 [0.24; 0.47]	0.08 [−0.05; 0.20]
BPQ42	−0.02 [−0.11; 0.07]	0.01 [−0.12; 0.13]	0.08 [−0.04; 0.20]	−0.05 [−0.14; 0.05]	**0.72 [0.63; 0.81]**	−0.02 [−0.09; 0.06]
BPQ43	−0.13 [−0.28; 0.02]	0.26 [0.12; 0.39]	0.08 [−0.06; 0.22]	0.10 [−0.05; 0.25]	0.17 [0.04; 0.30]	0.14 [0.01; 0.27]
BPQ44	−0.04 [−0.15; 0.07]	−0.09 [−0.23; 0.06]	−0.01 [−0.14; 0.11]	0.05 [−0.05; 0.16]	**0.77 [0.68; 0.85]**	0.01 [−0.07; 0.09]
BPQ45	−0.02 [−0.10; 0.07]	0.00 [−0.11; 0.11]	−0.14 [−0.27; −0.01]	0.09 [−0.01; 0.19]	**0.84 [0.75; 0.92]**	0.05 [−0.03; 0.14]
BPQ46	0.08 [−0.04; 0.19]	−0.07 [−0.21; 0.06]	0.03 [−0.09; 0.16]	−0.01 [−0.12; 0.10]	**0.41 [0.32; 0.51]**	0.13 [0.01; 0.24]
ρ with F2	0.40 [0.24; 0.55]					
ρ with F3	0.41 [0.30; 0.53]	0.05 [−0.18; 0.28]				
ρ with F4	0.28 [0.11; 0.45]	0.13 [−0.11; 0.37]	0.44 [0.31; 0.58]			
ρ with F5	0.31 [0.17; 0.45]	0.16 [−0.08; 0.39]	0.27 [0.11; 0.43]	0.40 [0.27; 0.52]		
ρ with F6	0.27 [0.05; 0.50]	0.11 [-0.28; 0.51]	0.34 [0.19; 0.50]	0.33 [0.20; 0.46]	0.34 [0.22; 0.46]	

Note: Bracketed values and the 95% confidence interval of the loading estimate. Bold values indicate that this interval is entirely over |0.32|.

**Table 4:** Goodness-of-fit indices for the confirmatory factor analysis models on the second random subsample (*n* = 693).

Model	χ^2^	df	CFI	TLI	RMSEA [90% CI]
One-factor	1841.033	350	0.788	0.771	0.078 [0.075; 0.082]
Three independent factors	3306.201	350	0.579	0.545	0.110 [0.107; 0.114]
Three correlated factors	872.283	347	0.925	0.918	0.047 [0.043; 0.051]
Bifactor	703.521	322	0.946	0.936	0.041 [0.037; 0.046]

Note: all chi-squared tests were significant at *p* < 0.001; df = degrees of freedom; CFI = comparative fit index; TLI = Tucker–Lewis index; RMSEA = root-mean-square error of approximation; CI = confidence interval.

**Table 5:** Parameter estimates of the one-factor (left), of the three-correlated-factor model (center), and of the bifactor model (right) (data from the second random subsample, *n* = 693).

Item	F1	F1	F2	F3	General	S1	S2	S3
BPQ04	**0.53**	**0.61**	-	-	**0.52**	**0.25**	-	-
BPQ05	**0.45**	**0.53**	-	-	**0.39**	**0.38**	-	-
BPQ12	**0.61**	**0.70**	-	-	**0.53**	**0.44**	-	-
BPQ15	**0.52**	**0.62**	-	-	**0.37**	**0.62**	-	-
BPQ16	**0.45**	**0.55**	-	-	**0.31**	**0.60**	-	-
BPQ17	**0.65**	**0.76**	-	-	**0.54**	**0.57**	-	-
BPQ18	**0.49**	**0.58**	-	-	**0.38**	**0.49**	-	-
BPQ19	**0.47**	**0.54**	-	-	**0.44**	**0.28**	-	-
BPQ24	**0.58**	**0.66**	-	-	**0.57**	**0.27**	-	-
BPQ25	**0.59**	**0.67**	-	-	**0.58**	**0.26**	-	-
BPQ07	**0.53**	-	**0.65**	-	**0.46**	-	**0.41**	-
BPQ10	**0.39**	-	**0.48**	-	**0.37**	-	**0.24**	-
BPQ13	**0.71**	-	**0.84**	-	**0.65**	-	**0.43**	-
BPQ14	**0.62**	-	**0.75**	-	**0.53**	-	**0.50**	-
BPQ42	**0.50**	-	**0.63**	-	**0.37**	-	**0.54**	-
BPQ44	**0.47**	-	**0.60**	-	**0.34**	-	**0.54**	-
BPQ45	**0.55**	-	**0.72**	-	**0.38**	-	**0.74**	-
BPQ46	**0.32**	-	**0.42**	-	**0.24**	-	**0.39**	-
BPQ27	**0.67**	-	-	**0.76**	**0.59**	-	-	**0.54**
BPQ28	**0.53**	-	-	**0.61**	**0.50**	-	-	**0.36**
BPQ30	**0.50**	-	-	**0.57**	**0.50**	-	-	**0.26**
BPQ32	**0.65**	-	-	**0.74**	**0.64**	-	-	**0.36**
BPQ33	**0.65**	-	-	**0.74**	**0.54**	-	-	**0.64**
BPQ34	**0.38**	-	-	**0.43**	**0.42**	-	-	**0.04**
BPQ35	**0.41**	-	-	**0.48**	**0.42**	-	-	**0.18**
BPQ37	**0.49**	-	-	**0.58**	**0.35**	-	-	**0.68**
BPQ38	**0.56**	-	-	**0.63**	**0.61**	-	-	**0.08**
BPQ39	**0.59**	-	-	**0.68**	**0.59**	-	-	**0.29**
ρ with F2		**0.48**						
ρ with F3		**0.62**	**0.56**					

Note: F = factor; S = specific factor; bolded values indicate that the estimate is significant at *p* < 0.001.

**Table 6:** Spearman correlations among body perception questionnaire (BPQ)-22 scales and the other measures employed in this study (data from the whole sample, *n* = 1361).

Scales	1	2	3	4	5	6	7	8	9	10	11	12
BPQ-BOA	*0.79*											
BPQ-SUP	0.38	*0.78*										
BPQ-BOA/SUB	0.39	0.42	*0.77*									
DASS-ANX	0.23	0.43	0.32	*0.83*								
DASS-DEP	0.12	0.26	0.28	0.61	*0.91*							
DASS-STR	0.18	0.34	0.36	0.66	0.75	*0.90*						
MSPQ	0.27	0.48	0.54	0.60	0.46	0.58	*0.82*					
OBQ-THR	0.14	0.35	0.21	0.43	0.49	0.49	0.39	*0.80*				
OBQ-RES	0.14	0.27	0.15	0.33	0.37	0.39	0.30	0.70	*0.84*			
OBQ-CON	0.08	0.25	0.11	0.36	0.34	0.35	0.28	0.66	0.63	*0.83*		
OBQ-PER	0.19	0.28	0.19	0.37	0.45	0.46	0.32	0.71	0.72	0.58	*0.85*	
SSAS	0.36	0.39	0.40	0.37	0.31	0.40	0.48	0.37	0.29	0.23	0.32	*0.72*
M	22.56	13.61	19.17	10.28	12.16	15.5	19.69	11.29	12.59	9.28	13.25	25.86
SD	6.87	3.88	5.3	3.8	5.11	5.19	5.51	5.58	6.13	5.13	6.84	5.82
Med	20.0	14.0	17.0	9.00	11.00	15.0	18.0	10.0	11.0	7.0	11.0	26.0
IQR	8	6	7	5	7	8	7	7	9	6	11	8

Note: correlations larger than |0.089| are significant at *p* < 0.001. Italicized values on the main diagonal are Cronbach’s alphas. BPQ-BOA: body perception questionnaire—body awareness; BPQ-SUP: BPQ-supradiaphragmatic reactivity; BPQ-BOA/SUB: BPQ-BOA/subdiaphragmatic reactivity; DASS-ANX: depression-anxiety-stress scale—anxiety; DASS-DEP: DASS—depression; DASS-STR: DASS—stress; MSPQ: modified somatic perceptions questionnaire; OBQ-THR: obsessive beliefs questionnaire—threat; OBQ-RES: OBQ-inflated responsibility; OBQ-CON: OBQ-importance and control of thoughts; OBQ-PER: OBQ-perfectionism/intolerance for uncertainty; SSAS: somatosensory amplification scale; M: mean; SD: standard deviation; Med: median; IQR: interquartile range.

**Table 7:** Test–retest results for the BPQ-22 scales (data from the second sample, *n* = 97).

Scale	M(Med)	SD(IQR)	M(Med)	SD(IQR)	*ρ*_tt_	ICC
BOA/SUB	16.76(15.50)	3.64(6)	17.27(16.00)	3.46(6)	0.77 ***	0.86 ***
SUP	12.52(11.00)	2.93(4)	12.94(10.00)	3.07(3)	0.74 ***	0.87 ***
BOA	19.54(18.00)	4.53(7)	20.28(19.00)	3.92(6)	0.78 ***	0.90 ***

Note: BOA/SUB: body awareness/subdiaphragmatic reactivity; SUP: supradiaphragmatic reactivity; BOA: body awareness; M: mean; Med: median; SD: standard deviation; IQR: interquartile range; *ρ*_tt_: Spearman’s rho test–retest correlation; ICC: intraclass correlation coefficient; ***: *p* < 0.001.

## Data Availability

The data presented in this study are available on request from the corresponding author. The data are not publicly available due to privacy issue.

## Supplementary Materials

The following are available online at https://www.mdpi.com/article/10.3390/ijerph18073835/s1, Table S1: Item scores distribution in the 5- and 3-point scoring conditions; Table S2: Loading matrix and factor correlations of the five-factor Exploratory Structural Equation Modeling solution; Table S3: Estimated marginal means (EMM) for Relationship Status categories in the BOA/SUB scale; Table S4: Post-hoc comparisons for Relationship Status categories in the BOA/SUB scale; Table S5: Estimated marginal means (EMM) for Relationship Status categories in the SUP scale; Table S6: Post-hoc comparisons for Relationship Status categories in the SUP scale; Body Perception Questionnaire—22 (BPQ-22); Correspondence of BPQ-22 items with the 46-item original English version.
