# Supplementary material for: Item Reduction, Psychometric and Biometric Properties of the Italian Version of the Body Perception Questionnaire—Short Form (BPQ-SF): The BPQ-22
Source: Int J Environ Res Public Health. 2021 Apr 6;18(7):3835. doi: 10.3390/ijerph18073835 (PMC8038843; doi:10.3390/ijerph18073835)
Supplement: Supplementary file 1 [file ijerph-18-03835-s001.pdf]

Supplementary Materials

Table S1.

Item scores distribution in the 5- and 3-point scoring conditions.

	5-point scoring					3-point scoring			
Item	1	2	3	4	5	1	2	3	Miss
bpq01	.28	.39	.24	.07	.02	.28	.39	.33	.00
bpq02	.16	.45	.25	.12	.03	.16	.45	.39	.01
bpq03	.18	.43	.25	.12	.03	.18	.43	.39	.01
bpq04	.18	.31	.30	.18	.03	.18	.31	.51	.01
bpq05	.17	.33	.26	.18	.07	.17	.33	.50	.01
bpq06	.07	.32	.29	.27	.05	.07	.32	.62	.01
bpq07	.09	.21	.31	.31	.07	.09	.21	.70	.01
bpq08	.12	.39	.28	.14	.07	.12	.39	.49	.00
bpq09	.22	.32	.25	.18	.03	.22	.32	.46	.01
bpq10	.32	.25	.24	.15	.04	.32	.25	.43	.00
bpq11	.39	.29	.20	.10	.02	.39	.29	.32	.00
bpq12	.12	.37	.29	.14	.08	.12	.37	.51	.01
bpq13	.08	.33	.29	.21	.08	.08	.33	.59	.01
bpq14	.16	.30	.28	.21	.05	.16	.30	.54	.01
bpq15	.49	.25	.14	.09	.04	.49	.25	.27	.00
bpq16	.48	.29	.12	.07	.04	.48	.29	.23	.01
bpq17	.72	.18	.06	.03	.03	.72	.18	.11	.00
bpq18	.09	.37	.30	.18	.05	.09	.37	.53	.01
bpq19	.32	.36	.20	.11	.02	.32	.36	.32	.00
bpq20	.40	.24	.20	.14	.03	.40	.24	.36	.01
bpq21	.03	.27	.37	.27	.06	.03	.27	.70	.00
bpq22	.74	.16	.08	.01	.01	.74	.16	.11	.00
bpq23	.07	.43	.34	.13	.03	.07	.43	.50	.00
bpq24	.48	.34	.12	.05	.01	.48	.34	.18	.01
bpq25	.08	.39	.34	.15	.04	.08	.39	.52	.01
bpq26	.26	.36	.22	.13	.03	.26	.36	.38	.00
bpq27	.75	.17	.06	.02	.01	.75	.17	.08	.00
bpq28	.67	.22	.08	.03	.01	.67	.22	.12	.01
bpq29	.38	.39	.16	.06	.00	.38	.39	.23	.00
bpq30	.72	.23	.05	.01	.00	.72	.23	.06	.00
bpq31	.22	.49	.22	.06	.00	.22	.49	.29	.00
bpq32	.61	.27	.09	.02	.00	.61	.27	.12	.00
bpq33	.79	.16	.04	.01	.00	.79	.16	.05	.00
bpq34	.80	.16	.03	.01	.00	.80	.16	.04	.00
bpq35	.88	.09	.02	.00	.00	.88	.09	.03	.00
bpq36	.57	.34	.08	.01	.00	.57	.34	.09	.01
bpq37	.89	.09	.02	.01	.00	.89	.09	.02	.01
bpq38	.66	.28	.04	.01	.00	.66	.28	.06	.01
bpq39	.54	.32	.10	.03	.01	.54	.32	.14	.00
bpq40	.85	.11	.03	.01	.00	.85	.11	.05	.01

bpq41	.60	.32	.06	.01	.00	.60	.32	.08	.01
bpq42	.26	.45	.16	.11	.02	.26	.45	.30	.01
bpq43	.34	.38	.15	.10	.03	.34	.38	.28	.00
bpq44	.46	.38	.13	.03	.00	.46	.38	.16	.00
bpq45	.22	.49	.18	.10	.02	.22	.49	.29	.01
bpq46	.26	.50	.16	.07	.00	.26	.50	.23	.00

Table S2.

Loading matrix and factor correlations of the five-factor Exploratory Structural Equation Modeling solution.

Item	F1	F2	F3	F4	F5
BPQ01	0.37 [0.23; 0.51]	0.39 [0.25; 0.52]	-0.04 [-0.13; 0.05]	0.03 [-0.06; 0.12]	-0.25 [-0.39; -0.11]
BPQ02	0.38 [0.24; 0.51]	0.33 [0.19; 0.47]	0.04 [-0.05; 0.13]	-0.11 [-0.23; 0.02]	-0.22 [-0.34; -0.09]
BPQ03	**0.44 [0.35; 0.54]**	0.24 [0.13; 0.34]	0.08 [-0.03; 0.18]	0.12 [0.00; 0.24]	-0.03 [-0.14; 0.08]
BPQ04	**0.47 [0.36; 0.58]**	0.10 [-0.01; 0.21]	-0.06 [-0.17; 0.04]	0.19 [0.07; 0.31]	0.15 [0.00; 0.29]
BPQ05	**0.58 [0.47; 0.68]**	-0.06 [-0.18; 0.05]	-0.07 [-0.19; 0.05]	0.03 [-0.07; 0.13]	0.12 [-0.03; 0.28]
BPQ06	0.43 [0.30; 0.56]	-0.06 [-0.17; 0.05]	0.20 [0.06; 0.34]	0.17 [0.04; 0.31]	0.02 [-0.09; 0.13]
BPQ07	0.17 [-0.04; 0.37]	-0.06 [-0.16; 0.04]	0.48 [0.31; 0.65]	0.40 [0.25; 0.54]	-0.05 [-0.16; 0.06]
BPQ08	**0.44 [0.32; 0.56]**	0.04 [-0.07; 0.15]	0.24 [0.12; 0.37]	-0.03 [-0.13; 0.08]	-0.09 [-0.22; 0.04]
BPQ09	0.40 [0.27; 0.53]	0.05 [-0.05; 0.14]	0.18 [0.05; 0.31]	0.19 [0.07; 0.31]	0.10 [-0.03; 0.23]
BPQ10	0.09 [-0.07; 0.24]	0.03 [-0.07; 0.13]	0.32 [0.17; 0.47]	0.37 [0.25; 0.49]	-0.03 [-0.14; 0.08]
BPQ12	**0.53 [0.38; 0.67]**	-0.15 [-0.26; -0.04]	0.05 [-0.06; 0.16]	0.05 [-0.05; 0.16]	0.24 [0.08; 0.39]
BPQ13	0.23 [0.04; 0.42]	-0.08 [-0.17; 0.01]	**0.67 [0.55; 0.78]**	0.06 [-0.05; 0.17]	0.08 [-0.03; 0.18]
BPQ14	0.13 [-0.07; 0.33]	0.01 [-0.05; 0.07]	**0.69 [0.55; 0.82]**	0.24 [0.11; 0.36]	-0.09 [-0.20; 0.02]
BPQ15	**0.60 [0.50; 0.70]**	0.00 [-0.09; 0.09]	-0.01 [-0.11; 0.09]	-0.12 [-0.23; 0.00]	0.12 [-0.03; 0.26]
BPQ16	**0.51 [0.41; 0.60]**	0.04 [-0.06; 0.14]	0.03 [-0.07; 0.13]	-0.16 [-0.29; -0.03]	-0.07 [-0.19; 0.05]
BPQ17	**0.68 [0.57; 0.79]**	0.16 [0.03; 0.30]	-0.06 [-0.17; 0.04]	0.00 [-0.09; 0.09]	0.08 [-0.06; 0.23]
BPQ18	**0.51 [0.41; 0.61]**	-0.07 [-0.18; 0.04]	0.10 [-0.02; 0.21]	-0.08 [-0.19; 0.04]	0.02 [-0.10; 0.13]
BPQ19	**0.55 [0.46; 0.64]**	0.04 [-0.06; 0.14]	0.01 [-0.08; 0.10]	-0.13 [-0.25; -0.02]	0.07 [-0.06; 0.21]
BPQ20	0.28 [0.17; 0.39]	0.12 [0.01; 0.24]	0.09 [-0.03; 0.20]	0.10 [-0.01; 0.22]	0.06 [-0.06; 0.18]
BPQ21	0.28 [0.11; 0.46]	-0.03 [-0.12; 0.06]	0.24 [0.11; 0.38]	0.06 [-0.06; 0.17]	0.36 [0.22; 0.50]
BPQ22	**0.48 [0.34; 0.61]**	0.01 [-0.11; 0.12]	0.16 [0.01; 0.32]	0.10 [-0.05; 0.24]	-0.03 [-0.15; 0.10]
BPQ23	0.40 [0.26; 0.55]	0.02 [-0.07; 0.11]	0.15 [0.02; 0.27]	0.10 [-0.02; 0.22]	0.26 [0.12; 0.39]
BPQ24	**0.50 [0.40; 0.60]**	**0.43 [0.32; 0.54]**	-0.04 [-0.12; 0.05]	-0.07 [-0.17; 0.04]	-0.09 [-0.22; 0.05]
BPQ25	0.46 [0.25; 0.68]	0.05 [-0.05; 0.14]	-0.01 [-0.07; 0.05]	-0.07 [-0.16; 0.03]	**0.62 [0.46; 0.78]**
BPQ27	-0.04 [-0.12; 0.04]	**0.51 [0.35; 0.66]**	0.10 [-0.01; 0.22]	**0.48 [0.35; 0.60]**	0.04 [-0.07; 0.14]
BPQ28	0.15 [0.04; 0.26]	**0.51 [0.39; 0.63]**	-0.05 [-0.17; 0.07]	0.25 [0.12; 0.37]	-0.01 [-0.11; 0.09]
BPQ29	0.10 [-0.08; 0.29]	0.20 [0.06; 0.34]	0.01 [-0.07; 0.08]	-0.04 [-0.13; 0.05]	**0.55 [0.42; 0.68]**
BPQ30	0.04 [-0.07; 0.14]	**0.61 [0.50; 0.71]**	0.08 [-0.04; 0.19]	0.02 [-0.09; 0.12]	0.06 [-0.06; 0.18]
BPQ31	0.05 [-0.06; 0.16]	0.38 [0.26; 0.49]	0.07 [-0.03; 0.16]	0.04 [-0.06; 0.13]	0.42 [0.30; 0.54]
BPQ32	0.04 [-0.04; 0.11]	**0.57 [0.43; 0.71]**	0.00 [-0.08; 0.07]	0.37 [0.25; 0.49]	0.18 [0.07; 0.30]
BPQ33	-0.05 [-0.12; 0.03]	**0.74 [0.60; 0.88]**	-0.01 [-0.09; 0.06]	**0.48 [0.35; 0.60]**	0.01 [-0.06; 0.09]
BPQ34	0.05 [-0.08; 0.17]	**0.60 [0.47; 0.73]**	0.05 [-0.07; 0.17]	-0.20 [-0.36; -0.05]	-0.13 [-0.30; 0.04]
BPQ35	0.01 [-0.09; 0.10]	**0.77 [0.67; 0.88]**	-0.06 [-0.17; 0.06]	-0.05 [-0.17; 0.07]	0.02 [-0.09; 0.14]
BPQ36	0.02 [-0.06; 0.10]	0.31 [0.18; 0.44]	0.20 [0.09; 0.32]	-0.15 [-0.27; -0.03]	0.29 [0.15; 0.43]
BPQ37	-0.13 [-0.25; -0.01]	**0.72 [0.60; 0.83]**	0.01 [-0.10; 0.11]	0.05 [-0.08; 0.18]	0.08 [-0.06; 0.23]
BPQ38	0.14 [0.02; 0.26]	**0.62 [0.52; 0.71]**	0.02 [-0.07; 0.11]	-0.03 [-0.13; 0.07]	-0.07 [-0.18; 0.05]
BPQ39	-0.06 [-0.14; 0.03]	**0.51 [0.39; 0.64]**	0.06 [-0.04; 0.16]	0.09 [-0.02; 0.21]	0.33 [0.20; 0.45]
BPQ40	0.17 [0.02; 0.31]	0.31 [0.16; 0.46]	0.11 [-0.04; 0.26]	-0.17 [-0.32; -0.02]	0.05 [-0.10; 0.20]
BPQ41	0.12 [0.01; 0.24]	0.19 [0.07; 0.31]	0.36 [0.24; 0.49]	-0.11 [-0.23; 0.02]	0.04 [-0.08; 0.15]
BPQ42	-0.05 [-0.19; 0.09]	0.04 [-0.05; 0.13]	**0.73 [0.65; 0.82]**	-0.08 [-0.19; 0.02]	-0.02 [-0.11; 0.06]

BPQ43	-0.01 [-0.11; 0.09]	0.14 [0.01; 0.26]	0.20 [0.09; 0.31]	0.17 [0.05; 0.28]	0.08 [-0.04; 0.19]
BPQ44	-0.17 [-0.33; 0.00]	0.09 [-0.01; 0.19]	**0.77 [0.68; 0.86]**	-0.09 [-0.20; 0.02]	0.03 [-0.05; 0.11]
BPQ45	-0.17 [-0.36; 0.01]	0.02 [-0.04; 0.08]	**0.85 [0.76; 0.94]**	0.02 [-0.04; 0.08]	0.09 [0.00; 0.18]
BPQ46	0.02 [-0.08; 0.11]	0.06 [-0.05; 0.17]	0.41 [0.31; 0.52]	-0.11 [-0.21; 0.00]	0.13 [0.01; 0.25]
ρ with F2	0.34 [0.22; 0.47]				
ρ with F3	0.35 [0.22; 0.49]	0.38 [0.26; 0.50]			
ρ with F4	0.11 [-0.02; 0.25]	-0.01 [-0.16; 0.15]	0.16 [0.02; 0.30]		
ρ with F5	0.26 [0.09; 0.43]	0.29 [0.16; 0.42]	0.36 [0.22; 0.50]	0.18 [0.05; 0.32]	

Note: Bracketed values and the 95% confidence interval of the loading estimate. Bolded values indicate that this interval is entirely over |.32|.

Table S3.

Estimated marginal means (EMM) for Relationship Status categories in the BOA/SUB scale.

Category	n	EMM	SE	lower.CL	upper.CL	HS
Divorced	65	16.64	0.70	15.27	18.01	a
Partner	633	15.70	0.36	14.99	16.40	a
Single	653	16.32	0.34	15.66	16.98	a
Widowed	10	12.68	1.62	9.51	15.85	a

Note: n: group size; SE: standard error; lower.CL and upper.CL: lower and upper 95% confidence limit, respectively; HS: Homogeneous subgroup (EEMs of categories that share a letter are not statistically different)

Table S4.

Post-hoc comparisons for Relationship Status categories in the BOA/SUB scale.

Contrast	Estimate	SE	df	t	p	adj-p	d
Divorced - Partner	0.94	0.64	1319	1.48	.140	.451	0.14 [-0.12; 0.40]
Divorced - Single	0.32	0.67	1319	0.48	.634	.964	0.05 [-0.21; 0.30]
Divorced - Widowed	3.96	1.67	1319	2.37	.018	.083	0.19 [-0.48; 0.86]
Partner - Single	-0.62	0.29	1319	-2.12	.034	.148	0.12 [0.01; 0.23]
Partner - Widowed	3.02	1.59	1319	1.90	.058	.230	0.42 [-0.20; 1.05]
Single - Widowed	3.64	1.61	1319	2.25	.024	.109	0.51 [-0.12; 1.13]

Note: SE: standard error; df: degrees of freedom; p: unadjusted *p* values; adj-p: *p* values after adjustment for false discovery rate for a family of 4 estimates; d: effect size *d* and its 95% confidence interval.

Table S5.

Estimated marginal means (EMM) for Relationship Status categories in the SUP scale.

Category	n	EMM	SE	lower.CL	upper.CL	HS
Divorced	65	10.76	0.49	9.79	11.73	ab
Partner	633	10.88	0.26	10.38	11.39	a
Single	653	11.48	0.24	11.01	11.96	b
Widowed	10	9.24	1.15	6.98	11.50	ab

Note: n: group size; SE: standard error; lower.CL and upper.CL: lower and upper 95% confidence limit, respectively; HS: Homogeneous subgroup (EEMs of categories that share a letter are not statistically different)

Table S6.

Post-hoc comparisons for Relationship Status categories in the SUP scale.

Contrast	Estimate	SE	df	t	p	adj-p	d
Divorced - Partner	-0.12	0.45	1330	-0.27	.784	.993	0.03 [-0.23; 0.28]
Divorced - Single	-0.73	0.48	1330	-1.53	.127	.421	0.15 [-0.11; 0.40]
Divorced - Widowed	1.52	1.19	1330	1.28	.201	.575	0.10 [-0.56; 0.77]
Partner - Single	-0.60	0.21	1330	-2.89	.004	.020	0.16 [0.05; 0.27]
Partner - Widowed	1.65	1.13	1330	1.45	.146	.466	0.32 [-0.30; 0.95]
Single - Widowed	2.25	1.15	1330	1.96	.051	.205	0.44 [-0.19; 1.06]

Note: SE: standard error; df: degrees of freedom; p: unadjusted *p* values; adj-p: *p* values after adjustment for false discovery rate for a family of 4 estimates; d: effect size *d* and its 95% confidence interval.

Body Perception Questionnaire – 22 (BPQ-22)

Italian version by**:**

Andrea Poli, Angelo Giovanni Icro Maremmani, Carlo Chiorri, Gian-Paolo Mazzoni, Graziella Orrù, Jacek Kolacz, Stephen W. Porges, Ciro Conversano, Angelo Gemignani, Mario Miccoli

Per favore, valuti il suo livello di consapevolezza per ciascuna delle caratteristiche descritte di seguito. Selezioni la risposta che la descrive più precisamente.

Nella maggior parte delle situazioni sono consapevole di:

		Mai	Occasionalmente	Spesso
1	Avere gli occhi inumiditi o che stanno lacrimando	❑	❑	❑
2	Una sensazione di gonfiore del mio corpo o di parti del mio corpo	❑	❑	❑
3	Avere la pelle d’oca	❑	❑	❑
4	Dolori di stomaco e intestino	❑	❑	❑
5	Un senso di gonfiore o dilatazione dello stomaco	❑	❑	❑
6	Sentire i palmi delle mani sudati	❑	❑	❑
7	Sudorazione della fronte	❑	❑	❑
8	Tremore delle labbra	❑	❑	❑
9	Sudorazione delle ascelle	❑	❑	❑
10	Quale sia la temperatura del viso (in particolare delle orecchie)	❑	❑	❑

Il sistema nervoso autonomo è la parte del suo sistema nervoso che regola il suo sistema cardiovascolare, respiratorio, digestivo e di regolazione della temperatura corporea. Inoltre, è coinvolto nell’esperienza e nell’espressione delle emozioni. Il sistema nervoso autonomo, in ognuno di noi, può funzionare in maniera diversa. Questa scala è stata sviluppata per misurare il modo in cui reagisce il suo sistema nervoso autonomo.

Per favore, valuti quanto ciascuna delle seguenti affermazioni sia frequente per lei:

11	Ho difficoltà a coordinare la respirazione mentre mangio	❑	❑	❑
12	Quando mangio, ho difficoltà a parlare	❑	❑	❑
13	Ho difficoltà a coordinare la respirazione con il parlare	❑	❑	❑
14	Quando mangio, ho difficoltà a coordinare la deglutizione, la masticazione e/o il succhiare con la respirazione	❑	❑	❑
15	Ho una tosse persistente che interferisce con il mio parlare e mangiare	❑	❑	❑
16	Mi sento soffocare quando mangio	❑	❑	❑
17	Quando parlo, spesso sento che dovrei tossire o deglutire la saliva che ho in bocca	❑	❑	❑
18	Quando respiro, sento di non riuscire ad avere abbastanza ossigeno	❑	❑	❑
19	Ho acidità di stomaco	❑	❑	❑

20	Ho un’indigestione	❑	❑	❑
21	Dopo aver mangiato ho problemi digestivi	❑	❑	❑
22	Ho la diarrea	❑	❑	❑

Scoring:

BOA: 1+3+6+7+8+9+10
SUP: 11+12+13+14+15+16+17+18
BOA/SUB: 2+4+5+19+20+21+22

Mai = 1; Occasionalmente = 2; Spesso = 3

Correspondence of BPQ-22 items with the 46-item original English version

Scale	Item # in the 46-item version	Item # in the 22-item version	Italian item	English item
BOA	5	1	Avere gli occhi inumiditi o che stanno lacrimando	Watering or tearing of my eyes
BOA/SUB	7	2	Una sensazione di gonfiore del mio corpo o di parti del mio corpo	A swelling of my body or parts of my body
BOA	12	3	Avere la pelle d'oca	Goose bumps
BOA/SUB	13	4	Dolori di stomaco e intestino	Stomach and gut pains
BOA/SUB	14	5	Un senso di gonfiore o dilatazione dello stomaco	Stomach distension or bloatedness
BOA	15	6	Sentire i palmi delle mani sudati	Palms sweating
BOA	16	7	Sudorazione della fronte	Sweat on my forehead
BOA	17	8	Tremore delle labbra	Tremor in my lips
BOA	18	9	Sudorazione delle ascelle	Sweat in my armpits
BOA	19	10	Quale sia la temperatura del viso (in particolare delle orecchie)	The temperature of my face (especially my ears)
SUP	27	11	Ho difficoltà a coordinare la respirazione mentre mangio	I have difficulty coordinating breathing and eating
SUP	28	12	Quando mangio, ho difficoltà a parlare	When I am eating, I have difficulty talking
SUP	32	13	Ho difficoltà a coordinare la respirazione con il parlare	I have difficulty coordinating breathing with talking
SUP	33	14	Quando mangio, ho difficoltà a coordinare la deglutizione, la masticazione e/o il succhiare con la respirazione	When I eat, I have difficulty coordinating swallowing, chewing, and/or sucking with breathing.
SUP	34	15	Ho una tosse persistente che interferisce con il mio parlare e mangiare	I have a persistent cough that interferes with my talking and eating
SUP	37	16	Mi sento soffocare quando mangio	I gag when I eat
SUP	38	17	Quando parlo, spesso sento che dovrei tossire o deglutire la saliva che ho in bocca	When I talk, I often feel I should cough or swallow the saliva in my mouth
SUP	39	18	Quando respiro, sento di non riuscire ad avere abbastanza ossigeno	When I breathe, I feel like I cannot get enough oxygen.
BOA/SUB	42	19	Ho acidità di stomaco	I have 'sour' stomach
BOA/SUB	44	20	Ho un'indigestione	I have indigestion
BOA/SUB	45	21	Dopo aver mangiato ho problemi digestivi	After eating I have digestive problems
BOA/SUB	46	22	Ho la diarrea	I have diarrhea

Note: BOA: Body Awareness; SUP: Supradiaphragmatic Reactivity; BOA/SUB: Body Awareness/Subdiaphragmatic Reactivity
